# Interprofessional peer-assisted learning for pharmacy and physical therapy students using inhalers and inhalation devices

**DOI:** 10.1186/s12909-023-04297-y

**Published:** 2023-05-02

**Authors:** C. Allyson Jones, Johnson Ching-hong Li, Mark Hall, Renette Bertholet, Tarek Turk, Cheryl A. Sadowski

**Affiliations:** 1 Department of Physical Therapy, Faculty of Rehabilitation Medicine, University of Alberta, Edmonton, AB Canada; 2 Department of Psychology, Faculty of Arts, University of Manitoba, Winnipeg, MB Canada; 3 Faculty of Pharmacy & Pharmaceutical Sciences, University of Alberta, Edmonton, AB Canada; 4 Faculty of Medicine and Dentistry, University of Alberta, Edmonton, Canada

**Keywords:** Interprofessional education, Physical therapy, Pharmacy, Peers, Inhalers

## Abstract

**Background:**

Peer-assisted learning (PAL) is exchanging knowledge between learners often from similar professional levels. Limited evidence exists on the effectiveness of PAL between different healthcare professions. The purpose of this study is to evaluate the knowledge, confidence, and perception of students engaged in an interprofessional PAL activity with pharmacy students instructing physical therapy students on the proper technique, cleaning/storage and therapeutic knowledge on inhaler devices for treatment of pulmonary conditions.

**Methods:**

Pharmacy and physical therapy students completed a survey before and immediately after the PAL activity. As instructors, pharmacy students rated their experience with inhalers, their confidence if they were to assist clients on the use of inhaler devices and confidence in teaching peers. Physical therapy students completed surveys on inhaler knowledge with 10 scenario-based multiple-choice questions, and their confidence if they were to assist clients with inhaler devices. The knowledge questions were grouped into three categories: storage and cleaning of inhalers (3 questions), technique of using inhalers (4 questions), and therapeutic knowledge of drugs given by inhalation (3 questions).

**Results:**

102 physical therapy and 84 pharmacy students completed the activity and surveys. For the physical therapy students, the mean improvement of the total score for knowledge-based questions was 3.6 ± 1.8 (p < 0.001). The question with the fewest number of correct answers (13%) before the PAL activity had the highest number of correct answers post-activity (95%). Prior to the activity, no physical therapy students felt *certain/very certain* about their knowledge on inhalers, yet after PAL activity this proportion increased to 35%. The percent of pharmacy students reporting their confidence as “certain” and “very certain” in teaching peers increased from 46% before the activity to 90% afterwards. Pharmacy students rated the monitoring and follow-up of inhaler devices as the lowest expectation for physical therapists to play a role. Steps taken to prepare for this PAL activity were also discussed.

**Conclusions:**

Interprofessional PAL can increase knowledge and confidence of healthcare students reciprocally learning and teaching in joint activities. Allowing such interactions facilitate students to build interprofessional relationships during their training, which can increase communication and collaboration to foster an appreciation for each other’s roles in clinical practice.

**Supplementary Information:**

The online version contains supplementary material available at 10.1186/s12909-023-04297-y.

## Background

Interprofessional education and learning (IPE) of healthcare students is regarded as a necessary approach to promote early and sustainable skills to effectively work in a healthcare team [1, 2]. The goal of IPE is for students to learn to become active members of a healthcare team while bringing their knowledge, skills and values of their discipline to the team [3]. The ultimate intention of an interprofessional healthcare team is to provide patient-centred care in a collaborative manner. Learning experiences focussing on a collaborative approach provide communication and interaction skills which are foundational for successful interprofessional teamwork in clinical settings [4]. Active learning is one approach of IPE that helps prepare students in healthcare for interprofessional collaboration [1, 5]. As practicing clinicians, active learning also nurtures career-learning so they can manage gaps in knowledge and skills by critical thinking and reflection [6]. One form of active learning approach is peer-assisted learning (PAL) which is defined as “*people from similar social groupings who are not professional teachers helping each other to learn and learning themselves by teaching*” [7]. Peer-assisted learning has more recently gained interest in medical and allied health education [8, 9] with such benefits as encouraging mutual understanding and collaboration, and helping to clarify professional roles [10, 11]. Aside from the traditional didactic instruction delivered by professors to students, PAL can improve theoretical knowledge and practical clinical skills [8, 12]. In a systematic review of 19 trials investigating the impact of peer-teaching activities on learning outcomes of medical students, PAL achieved short-term learner outcomes comparable to conventional teaching suggesting positive effects on student-teacher learning outcomes [12].

Evidence of PAL within healthcare has been primarily documented within a single discipline, yet limited work has examined the effectiveness of PAL between disciplines [10, 13–16]. Our earlier work [16] provided the basis to have undergraduate pharmacy students instruct entry-to-practice physical therapy students about the use of inhaler devices. Inhaler devices are commonly used to deliver medication to people with chronic respiratory conditions such as asthma and chronic obstructive pulmonary disease (COPD). Several types of devices exist for inhaled medication delivery. The diversity of products is primarily due to the Montreal Protocol, which called for the removal of CFC inhalers from the market by 1996 to reduce ozone-depleting substances [17]. A variety of alternate propellant and dry powder inhalers have since been developed. Using the correct technique for each of these inhalers is critical, as incorrect technique can increase adverse effects [18, 19]. Proper instruction by healthcare professionals, such as physicians, nurses, pharmacists, physical therapists and respiratory therapists, is recommended to avoid inhaler mishandling by patients [20]. Interprofessional collaboration and education in the proper use of inhalers and inhalation devices is essential to ensure consistency, monitoring, and supporting effective disease management.

Clinical guidelines by the National Heart, Lung, and Blood Institute recommend that inhaler technique be reviewed on a regular basis, and that self-management education involves an interprofessional approach by members of the healthcare team [21]. This includes the integral role of pharmacists in medication management and monitoring inhaler technique. Physical therapists also have important roles in the management of patients with respiratory diseases. Strategies of respiratory physical therapy are aimed at reducing the work of breathing, improving ventilation, increasing function, and enabling relief of dyspnea [22, 23]. To augment mucocilliary clearance techniques, coordination of the use of medications to clear secretions is a key component [23]. Within their cardiorespiratory physical therapy training, students learn the proper technique for use and cleaning/storage of inhaler devices.

Our earlier experience with a PAL activity evaluated whether physical therapy students teaching pharmacy students was an effective method to improve knowledge and skills of basic ambulatory devices that were commonly sold in community pharmacies [16]. Because this PAL activity was viewed as a positive experience by both instructors and students, an equitable approach was to have pharmacy students instruct physical therapy students on proper technique, cleaning/storage and therapeutic knowledge of inhaler devices. Our initial study focused only on the learner rather than the students who were in the teaching role [16]. Building upon our earlier work, the purpose of this current study is to evaluate the knowledge, confidence, and perception of students engaged in an interprofessional peer assisted learning activity with pharmacy students instructing physical therapy students on the proper technique, cleaning/storage and therapeutic knowledge on inhaler devices for treatment of pulmonary conditions.

## Methods

### Course context and curriculum delivery

At our institution, there is a strong educational culture of interprofessional learning within the health sciences that abide by the general principles of interprofessional education [24]. Because the pharmacy and physical therapy instructor teams had successfully collaborated on an earlier PAL activity [16], this inhaler single PAL activity was a logical follow-up. More importantly, these PAL activities ensured that students from both pharmacy and physical therapy had the opportunity to be in a teaching role.

The success of the PAL activity was also contingent upon logistical concerns such as developing the schedules/curriculum, finding common schedule times, and accommodating physical space [3]. Instructors from the pharmacy and physical therapy programs met to discuss the inaugural PAL activity a year before the actual activity so that changes could be made to the schedules/curriculum and booking of physical space. An initial step was to determine which courses this activity was to be housed in and then include the activity as a mandatory requirement in the respective course schedules. The pharmacy instructor team selected the inhaler teaching skills course to conduct the PAL activity, whereas the cardiorespiratory course was selected by the physical therapy team to learn this skill. Within the four-year undergraduate pharmacy program, second year students were taught therapeutic knowledge within their pulmonary course, and skills/simulation laboratory, of which students learned about inhaler device types, technique, maintenance of inhalers, and communication with patients in the academic terms prior to the PAL activity. The learning objective of the pharmacy PAL activity was to practice educating another healthcare profession, physical therapy about inhaler devices. Within the 28 months Master’s program, physical therapy students learned about inhalers in their first-year cardiorespiratory course. A small subset of physical therapy students (n = 18) who were at a satellite centre located in another city, received all of their courses including the cardiorespiratory course via video-conferencing. Physical therapy instructors were also located at this satellite site to help with coordination and organization of their courses. For the physical therapy students, the objectives of the PAL activity were to learn the correct method to deliver inhaled drugs using various devices and to gain experience learning with and from students from another healthcare profession. The preparation for both pharmacy and physical therapy students prior to the activity included a discussion in their respective courses to gain an understanding of each other’s role and how to work together across disciplines in clinical health teams [25, 26] with respect to management of inhalers.

An essential feature of the PAL activity was that standardized information be taught. In preparation of the activity, pharmacy students were assigned to a teaching group of 3 students. Each pharmacy group prepared handouts on inhaler devices that were to be given to the physical therapy students. These handouts were submitted to the instructor for review and the best designed handout, as evaluated by the course coordinator (RB), was used by all pharmacy groups. The standardized handout included images of the common inhalers used for chronic airway diseases, types of medications and their mechanism of action, medication side effects, basic techniques for all inhaler types currently available on the Canadian market cleaning/storage, cost and coverage, red flags, common administration challenges, and resources. After completing this activity, pharmacy students individually completed a written reflection exercise regarding their experiences. The instructor reviewed the teaching plans for accuracy and compiled a standardized plan for all pharmacy groups to use for this PAL activity. Pharmacy students were marked on their teaching plan assignment and their reflective journal entry, while physical therapy students were examined on their final written cardiorespiratory course exam about inhaler knowledge taught in the PAL activity.

A key logistical issue concerned accommodation of the large number of students given the availability of physical space. The 238 students were assigned to small groups (3 pharmacy:2 or 3 physical therapy students) and the PAL activity was divided into two 1-hour sessions of 21 groups each. Physical therapy students learning at the satellite location were also assigned to groups and the instruction occurred over video conference. They received the same equipment as the other student groups.

### Study assessment

To address any implementation issues, we piloted the inhaler PAL activity the year before with a different cohort of pharmacy and physical therapy students. Because no issues arose, a formal evaluation for the current cohort was implemented. A pre-post study design was executed for this evaluation. Pharmacy and physical therapy students were invited to participate based on announcements in their courses prior to the scheduled activity. Students were informed that the course activities were mandatory and part of the curriculum; however, as per ethics requirements, pre/post surveys could be completed if they consented to participate in this study. Students were provided with information sheets regarding the study and written consents were obtained. Ethics approval was secured from the Health Research Ethics Review Board of the University of Alberta (PRO00036759).

### Study procedures

Immediately before and after the PAL activity, consenting physical therapy students individually completed a paper-format survey which included demographics, 10 knowledge-based multiple-choice questions, a question regarding students’ confidence if they were to assist clients with inhaler devices, and perception of physical therapists’ role in assisting clients with inhalers. The knowledge questions for physical therapy students were grouped into three categories: storage and cleaning of inhalers (3 questions), technique of using inhalers (4 questions), and therapeutic knowledge of drugs given by inhalation (3 questions). All 10 questions were scenario-based (applied knowledge). The post-survey, which was completed immediately after the PAL activity, consisted of all the questions asked before the activity except the demographic information. Although the pre-post knowledge-based questions were the same, the order of questions varied.

Pharmacy students who provided consent, individually completed eight questions before the PAL activity, including demographics, past experience with use of different types of inhalers, confidence if they were to assist clients with inhaler devices, confidence in teaching and perception of physical therapists’ role in assisting clients with inhalers. The post-survey consisted of questions about the pharmacy students’ confidence if they were to assist clients with inhaler devices, confidence in teaching other healthcare professional students, and three open ended questions about the effectiveness of the PAL activity. All of the confidence ratings for pharmacy and physical therapy surveys used a 7 point Likert scale ranging from ‘*very uncertain’* (1) to *‘very certain’* (7).

### Analysis

Descriptive statistics were calculated for all variables. Scoring for the correct pre and post activity answers were completed for the 10 physical therapy knowledge-based questions. Using paired t-tests, total test scores were compared between pre- and post-test questions to assess improvement of physical therapy students. Statistical analyses were performed using SPSS Statistics version 24 software.

## Results

Of the 112 eligible physical therapy students, 107 answered the survey before the PAL activity and 102 answered the survey immediately after the activity, while 84 of 126 pharmacy students answered the survey before the activity and 91 afterward. The cohort characteristics are shown in Table 1. Seven additional pharmacy students who only completed the post-activity survey were excluded from the pre-post analysis. The majority of students from both disciplines were female. Physical therapy students were slightly older than the pharmacy students. The mean number of university years was 5.0 ± 1.1 years for physical therapy and 4.0 ± 1.3 for pharmacy students.

**Table 1:** Characteristics of pharmacy and physical therapy students who completed the survey before and after the peer-assisted learning activity

Characteristics	Pharmacy Students n = 84	Physical Therapy Students n = 107
Gender (female), n (%)	51 (60.7)	75 (70.1)
Age, n (%):		
21 years	37 (44.0)	0
22 years	13 (15.5)	16 (15.0)
23 years	15 (17.9)	27 (25.2)
24 years	4 (4.8)	27 (25.2)
25–29 years	13 (15.5)	34 (31.8)
30–34 years	2 (2.4)	3 (2.8)
Years of completed university education, mean ± SD	4.2 ± 1.3	5.0 ± 1.1
Education, Degrees completed prior to entering program, n (%):		
BA	1 (1.2)	10 (9.3)
BSc	21 (25.0)	67 (62.6)
Masters Degree	-	2 (1.9)
Other	2 (2.4)	28 (26.2)
No degree	60 (71.4)	-
Previous experience at a pharmacy, n (%)	63 (75.0)	-
Previous experience with inhalers, n (%):		
Metered dose inhaler	51 (60.7)	6 (5.6)
Spacer	27 (32.1)	1 (0.9)
Discus	26 (31.0)	3 (2.8)
Turbuhaler	29 (34.5)	0
Handihaler	13 (15.5)	1 (0.9)
Nebulizer	3 (3.6)	5 (4.7)

### Physical therapy students knowledge

Physical therapy students were asked 10 knowledge questions pre-post activity (Table 2). Out of 10 possible points, the mean scores were 3.8 ± 1.3 (range = 0 to7) pre-activity and 7.5 ± 1.3 (range = 3 to10) post-activity. The mean improvement of 3.6 ± 1.8 from pre-to-post-activity scores was significant (p < 0.001). The knowledge questions that were most frequently answered incorrectly before the PAL activity were (1) loading the appropriate dosage or form of medication with a Handihaler (87%, n = 93), and (2) the importance of rinsing the mouth after use (81%, n = 87). The question that had the most students who answered correctly was the type of rescue medication used in inhaler form (77%, n = 82). There was no one student who answered all 10 questions correctly while 28% (n = 30) of students had all incorrect answers. Interestingly, the question that had the fewest number of correct answers before the PAL activity, loading the appropriate dosage or form of medication with a Handihaler (13% correctly answered), had the highest number of correct answers (95%, n = 97) after the PAL activity. The post survey question that had the fewest number of correct responses (44%, n = 45) concerned properly cleaning a spacer. No differences were seen between the in-person and satellite cohorts.

**Table 2:** Physical therapy students scores for knowledge-based questions before and after peer-assisted learning activity

n = 102	Number of knowledge-based questions	Pre-score (mean ± SD) range of scores		Post-score (mean ± SD) range of scores
Total score*	10	3.8 ± 1.3 0–6	7.5 ± 1.3 3–10	
Subscale scores*:				
- Proper storage and cleaning of inhalers	3	1.1 ± 0.87 0–3	2.0 ± 0.76 0–3	
- Appropriate technique for using inhalers	4	1.0 ± 0.81 0–3	3.1 ± 0.84 1–4	
- General knowledge on inhalers	3	1.6 ± 0.71 0–3	2.4 ± 0.73 0–3	

* mean difference between pre & post scores < 0.001

### Students’ confidence and perception of roles

**Fig. 1:**
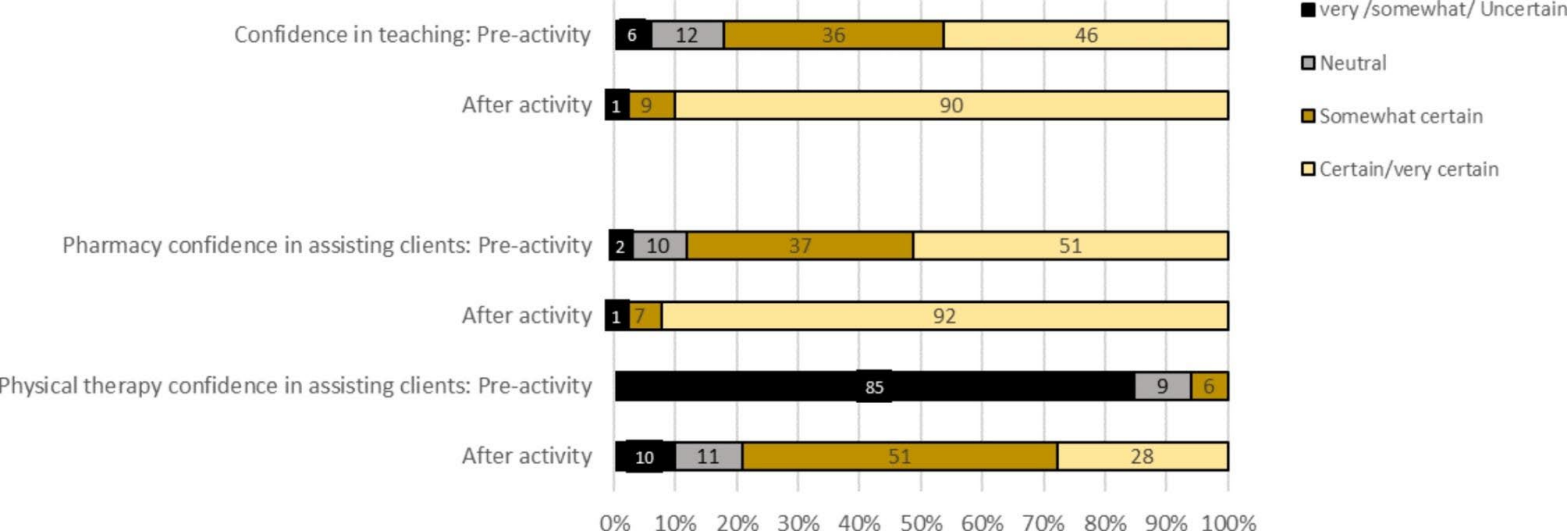
Pharmacy students’ self-rated confidence before and after peer-assisted learning activity with teaching other healthcare professionals. Pharmacy and physical therapy students’ self rated confidence of assisting clients with inhalers before and after the activity

Using a 7-point Likert scale, both disciplines were asked to rate their confidence if they were to assist clients with inhaler devices (Fig. 1). Before the activity, no physical therapy students were *certain/very* certain about their ability to assist clients with inhalers; however, this number increased to 28% after the activity. Pharmacy students also showed increased confidence post-activity. Before the activity, 51% pharmacy students felt *certain*/*very certain* in assisting a client with an inhaler, while 92% felt so afterward. Before the PAL activity, 46% of pharmacy students felt *certain/very certain* in teaching other health professionals, while 90% felt *certain/very certain* in teaching professionals after the activity. The majority of pharmacy students felt that physical therapists should be involved with teaching the correct technique, cleaning and storage, and assessing technique, yet 31% (n = 26) of pharmacy students felt physical therapists should be involved with monitoring safe and effective use of the medications and devices (Table 3). A proportion of physical therapy students (35%, n = 37) felt their role should include teaching cleaning and storage of inhaler devices.

**Table 3:** Students’ perceptions of physical therapists’ role for assisting clients with inhalers devices*

Perceived Role	Pharmacy Students n = 84 n(%)	Physical Therapy Students n = 102 n(%)
Teaching correct technique	71 (84)	61 (57)
Teaching cleaning and storage	58 (69)	37 (35)
Assessing technique	72 (86)	61 (57)
Monitoring safe and effective use of the medications and devices	26 (31)	68 (63)

* Survey question: What do you feel a physical therapist’s role should be in assisting clients with inhaler devices? Please check all that apply. Items are listed above in the table

## Discussion

Our findings highlight the importance and effectiveness of interprofessional PAL with pharmacy and physical therapy students. This aligns with previous studies that describe the effect of interdisciplinarity on newfound respect for the other profession, positive attitudes of students, and improved student confidence [10, 13]. Articles spanning the past 3 decades demonstrate that pharmacy and medical students involved in interprofessional education found the experiences useful, most favored more sessions, and interprofessional competency improved [14, 27, 28]. Another example involved medical and nursing students where all participants felt that interprofessional PAL activities improved their knowledge and confidence [15]. Our study highlights an important aspect of interprofessional PAL in that students who are in the teaching role also improve their confidence and knowledge regarding the subject matter. Besides physical therapy students increasing their knowledge, the pharmacy students demonstrated their clinical expertise, and practiced teaching skills which offered training in leadership, organizational skills, developing educational material for another profession, and effective group communications, all valuable traits for clinical practice. These skills practiced with this interprofessional PAL activity would have otherwise been missed if the instructors had taken a didactic teaching approach.

As recognized by others, a well-structured plan is needed for PAL activities [29]. This was exemplified by one knowledge-based question for physical therapy students concerning the correct sequence of devices to be used. Fewer students scored the correct answer on post-activity survey than before the activity. Upon assessing potential reasons, we found this topic was not equally highlighted in the standardized learning plan as the peer teachers focused more on technique and storage/cleaning of devices. To avoid this pitfall, a detailed learning plan with a checklist for teachers outlining key topics should be provided. Another interesting finding was the perception of physical therapist’s role with inhalers. Most of the pharmacy and physical therapy students regarded monitoring and teaching cleaning and storage of inhalers as not the role of physical therapists. Both pharmacy and physical therapy students did not have clinical experience at this period in their programs and it could be that they did not understand the extensive role of physical therapy in cardiorespiratory. This lends itself to a more comprehensive explanation each other’s role when the instructors are preparing students for the PAL activity.

Our cohort also included a subset of physical therapy students who video-conferenced from a different city, which was a different experience than the face-to-face interactions. Online learning brings several added values to the educational process such as increasing accessibility for students in remote areas and improving convenience for students who might attend from home [30, 31]. On the other hand, online learning has several reported challenges such as technical difficulties and the inability of providing proper instructor-supervised hands-on training [30, 31]. Pharmacy students noted that it was difficult to ensure that physical therapy students were using correct technique when they could view them by video only. These findings are particularly relevant as the COVID19 pandemic markedly affected learning when students worldwide had to shift to online platforms to continue their education. This highlights the need to adapt and further study online PAL activities for various topics in health education (e.g., practical versus theoretical skills), and in different settings (e.g., poor versus rich-resource settings).

Many challenges with implementation of peer teaching across health care professions were recognized in spite of our institution being supportive of interprofessional learning [3]. These issues centred primarily on the logistical planning of a PAL activity. Although this activity required coordinating with another instructor from another faculty beforehand, the outcomes were rewarding given the students’ knowledge gained and the positive informal feedback from students. The first step was to identify a topic from which students from different professions would benefit, and a topic where both professions could provide reciprocal teaching to each other [32, 33]. This involved research and discussion between the two interested faculties as to what topics students may benefit from and what peers can teach. In our case, inhaler technique for the treatment of pulmonary conditions was chosen as retention of the correct technique is a clinical challenge with up to 50% of patients failing to use inhalers correctly over time [18, 19], and as reinforcing patients’ knowledge about inhaler use is relevant and vital for both health professions. Another challenge included coordination between the two faculties to organize a time that was feasible within the curriculum, and finding the physical space to accommodate a large number of students [3, 32, 33]. To implement the activity smoothly, there was extensive planning and communication between the two faculties, instructors and lab assistants that were running the PAL activity. Another challenge was to ensure standardization of material taught, which involved having the students prepare learning plans ahead of time, and providing them with uniform objectives and activities during the PAL activity. To overcome this challenge, the student teachers needed to be trained and prepared for the session, with specific outcomes they were required to meet [34]. In this study, the students created a learning plan to create standardization, but they also could have been provided with a uniform learning plan, specific learning objectives, or an approximate timeline of activities.

The study findings provide support for the effectiveness of the interprofessional allied health professional PAL activity; however, non-participant bias and students who did not complete both before and after surveys warrant acknowledgement as possible limitations of this study. Due to ethics protocol, we could not identify who and why students chose not to participate in the assessment. Because a small number of students (n = 12) completed one survey and not the pre and post surveys, this likely would not have impacted the overall findings of this evaluation. Although the response rate was excellent (83%), the pre-post survey design was for a single cohort and further assessments of future student cohorts will provide a better understanding of these results within the context of allied health education.

## Conclusions

This study demonstrated that pharmacy and physical therapy students can increase knowledge and confidence with interprofessional PAL activity for inhaler devices to treat pulmonary conditions. Students were able to develop and practice teaching skills along with instilling professional values. Although it may be difficult to develop, organize and implement these activities, it provided a valuable experience for healthcare professional students to interact and promote collaboration between the professions. Allowing such interactions encourage students to build interprofessional relationships during their training, which in turn, can increase communication and collaboration to foster an appreciation for each other’s roles in clinical practice.

## Electronic supplementary material

Below is the link to the electronic supplementary material.

Supplementary Material 1

## Data Availability

The dataset supporting the conclusions of this study is available from the corresponding author upon reasonable request. The data cannot be publicly shared based on confidentiality agreements approved by the Human Research Ethics Committee regarding study participants.

## Abbreviations

COPD: chronic obstructive pulmonary disease
IPE: interprofessional education
PAL: peer-assisted learning
