# Supplementary material for: Interprofessional peer-assisted learning for pharmacy and physical therapy students using inhalers and inhalation devices
Source: BMC Med Educ. 2023 May 2;23:301. doi: 10.1186/s12909-023-04297-y (PMC10152630; doi:10.1186/s12909-023-04297-y)
Supplement: Supplementary file 1 — Supplementary Material 1 [file 12909_2023_4297_MOESM1_ESM.pdf]

PHARMACY Inhaler Devices
PRE-TEST EVALUATION

NAME (PRINTED): ____

Student ID Number:____

Knowledge and Confidence

1. Please rate your overall confidence in assisting clients with inhaler devices.

   a) 1 - Very Uncertain
   b) 2 - Uncertain
   c) 3 - Somewhat uncertain
   d) 4 - Neutral
   e) 5 - Somewhat certain
   f) 6 - Certain
   g) 7 - Very Certain

2. Please rate your overall confidence in teaching other health professionals or health professional students.

   a) 1 - Very Uncertain
   b) 2 - Uncertain
   c) 3 - Somewhat uncertain
   d) 4 - Neutral
   e) 5 - Somewhat certain
   f) 6 - Certain
   g) 7 - Very Certain

Demographic Information

1. a. Male b. Female

2. Age
    a. 19 or younger
    b. 21
    c. 22
    d. 23
    e. 24
    f. 25 or older

3. How many years of university have you completed?
    a. 3 yrs
    b. 4 yrs
    c. 5 yrs
    d. 6 yrs
    e. 7 yrs
    f. 8 yrs
    g. 9 or more

4. Did you complete a degree/degrees prior to Pharmacy?
    a. BA
    b. BSc
    c. Other
    d. No degree

Exposure to Inhaler Devices

5. Have you ever worked in a pharmacy as a pharmacy assistant outside of rotations?
    a. Yes.
    b. No.

6. If yes, have you ever assisted a patient/client in use of his/her inhaler device? Please check all that apply.

a. Metered dose inhaler
b. Spacer
c. Discus
d. Turbuhaler

e. Handihaler
f. Nebulizer
g. Not Applicable

Students' Perception of Role

7. What do you feel a physiotherapist's role should be in assisting clients with inhaler devices? Please check all that apply.

a. Teaching on correct inhalation technique
b. Teaching on cleaning/care of inhaler device
c. Assessing technique
d. Monitoring safe and effective use of the medications and devices
e. Other

PHYSICAL THERAPY Inhaler Devices
PRE-TEST EVALUATION

NAME (PRINTED): ____

Student ID Number:____

Please circle the best answer for the following multiple choice questions.

The next 6 questions are based on the following scenario.

Sumit, a 23-year old university student, has been diagnosed with asthma. He is prescribed a steroid inhaler, a bronchodilator (long-acting beta-agonist).

1. What is the correct sequence for use of the inhalers for scheduled dosing?
   a) Steroid, then bronchodilator
   b) Bronchodilator, then steroid
   c) Use both at the same time
   d) It does not matter which sequence is used

2. If Sumit uses a spacer he should clean it by:
   a. Putting on top rack of dishwashwer
   b. Bleaching it
   c. Washing with a special detergent provided with his inhaler prescription
   d. Using an abrasive brush

3. When Sumit uses the spacer appropriately he should:
   a. Hear a whistle
   b. Hear nothing
   c. See a change in colour inside the spacer
   d. See an increase in the pressure counter on the side of the spacer

4. If Sumit was using a metered-dose inhaler (MDI) and in winter wanted to participate in an outdoor activity we would instruct him to keep it:
   a. outside of his coat but accessible for use
   b. near his body in an internal pocket or around his neck
   c. in his backpack
   d. indoors – MDI's cannot be taken outside

5. Sumit is instructed to rinse his mouth with water after using his inhalers. This helps reduce:
   a. Dental caries
   b. Yeast infection
   c. Stomatitis
   d. Oral absorption of the medication

6. A rescue medication that Sumit would be prescribed is
   a. Salbutamol (Ventolin)
   b. Tiotropium (Spiriva)
   c. Budesonide (Pulmicort)
   d. Fluticasone/Salmeterol (Advair)

Emily is a 67 year old patient who was diagnosed with COPD recently. She is starting a new regimen of inhalers.

7. Emily is started on tiotropium, administered with the HandiHaler. This device is unique in that the patient has to load the medication as a:

    a. Powder
    b. Capsule
    c. Canister
    d. Disc

8. Emily is having some difficulty with the inhalers and often breathes out when she is holding the device. Which device is it okay to breathe into without affecting the medication?

    a. Metered dose inhaler
    b. Diskus
    c. Handihaler
    d. Turbuhaler

9. If Emily is assigned to use a Diskus inhaler we would want to warn her that she should keep it away from
    a. Sunlight
    b. Moisture
    c. Vibration
    d. Cold

10. Out of all the devices used, the one that requires the greatest inspiratory effort is
    a. Handihaler
    b. Turbuhaler
    c. Metered Dose Inhaler
    d. Diskus

Demographic Information

1. a. Male b. Female

2. Age
   a. 21 or younger
   b. 22
   c. 23
   d. 24
   e. 25 or older

3. How many years of university have you completed?
   a. 3 yrs
   b. 4 yrs
   c. 5 yrs
   d. 6 yrs
   e. 7 yrs
   f. 8 yrs
   g. 9 or more

4. Did you complete a degree/degrees prior to PTER?
   a. BA
   b. BSc
   c. Other
   d. No degree

Exposure to Inhaler Devices

5. Have you ever assisted a patient/client in use of his/her inhaler device? Please check all that apply.
   a. Metered dose inhaler
   b. Spacer
   c. Discus
   d. Turbuhaler
   e. Handihaler
   f. Nebulizer
   g. Not Applicable

Knowledge of Inhaler Devices

6. Please rate your overall confidence in assisting clients with inhaler devices.

   a. 1 - Very Uncertain
   b. 2 - Uncertain
   c. 3 - Somewhat uncertain
   d. 4 - Neutral
   e. 5 - Somewhat certain
   f. 6 - Certain
   g. 7 - Very Certain

Physiotherapy Students' Perception of Role

7. What do you feel a physiotherapist's role should be in assisting clients with inhaler devices? Please check all that apply.

   a. Teaching on correct inhalation technique
   b. Teaching on cleaning/care of inhaler device
   c. Assessing technique
   d. Monitoring safe and effective use of the medications and devices
   e. Other
